# Digital readiness and family support are associated with later learning engagement through digital self-efficacy among rural Chinese children

**DOI:** 10.1038/s41598-026-58145-2

**Published:** 2026-06-14

**Authors:** Xiang Yu, Ruixue Wu, Yidan Ma, Chunyan Jin

**Affiliations:** School of Education Science, Leshan Normal University, Leshan, China

**Keywords:** Digital readiness, Learning engagement, Digital self-efficacy, Family support, Rural children, Health care, Psychology, Psychology

## Abstract

Digital technologies are increasingly embedded in primary education, yet rural children’s engagement may depend not only on access to technology but also on readiness to use digital resources for learning. This two-wave time-lagged study examined whether digital readiness was associated with later learning engagement among rural Chinese children through digital self-efficacy, and whether family support strengthened this indirect association. Participants were 768 Grade 5 and Grade 6 students from 15 rural primary schools in Sichuan Province, China. Digital readiness, family support, baseline learning engagement, and demographic variables were assessed at T1; digital self-efficacy and learning engagement were assessed three months later at T2. After controlling for baseline engagement and demographic covariates, T1 digital readiness was positively associated with T2 learning engagement. Digital self-efficacy partially accounted for this longitudinal association, and family support strengthened the association between digital readiness and digital self-efficacy. Measurement validation, attrition effect sizes, and school-clustered robustness checks supported the robustness of the findings. These results suggest that rural children’s later learning engagement is linked to readiness-related conditions and family support, but the findings should be interpreted as longitudinal associations rather than causal effects.

**Supplementary Information:**

The online version contains supplementary material available at https://doi.org/10.1038/s41598-026-58145-2.

## Introduction

 As digital technologies continue to enter both school and home learning environments, children’s learning experiences are being reshaped in fundamental ways. Online platforms, digital resources, and smart devices have created more flexible opportunities for learning, but technology exposure does not automatically translate into meaningful educational benefits. Increasingly, the key question is not simply whether children have access to technology, but whether they are ready to use digital resources in ways that support sustained learning^1–3^. Recent international reports have similarly emphasized that children’s opportunities and well-being in the digital age depend not only on access, but also on the quality of their participation in digital environments (OECD^4^, UNICEF Innocenti^5^.

In many rural Chinese school contexts, this question is particularly relevant, although rural children should not be treated as a homogeneous group. Compared with students in more resource-rich settings, some rural students may have less stable digital learning environments, fewer forms of technical guidance, and more limited family educational resources. Evidence from Chinese primary-school samples suggests that rural students may score lower than urban students on information and communication technology (ICT) literacy and online learning self-efficacy^6^. This evidence does not imply a universal rural disadvantage, but it does suggest that digital inequality in children’s learning can involve not only material access, but also skills, confidence, and readiness for educational use of technology.

The broader digital-divide literature provides a useful framework for understanding this shift. Dijk^7^, conceptualized digital inequality as a layered process involving access, skills, use, and outcomes, and van Deursen and van Dijk^8^, further showed that when basic access becomes more widespread, inequalities increasingly shift toward differences in usage quality and digital skills. Livingstone and Helsper^9^, also highlighted the role of family mediation in children’s internet use, suggesting that children’s digital opportunities are shaped by social guidance as well as access. Together, these perspectives support a move from access-based digital inequality toward readiness-based digital inequality in educational contexts.

Against this background, digital readiness provides a learning-oriented lens for understanding why children in broadly similar digital environments may still differ in the quality of their learning participation. In this study, digital readiness is conceptualized as a study-specific adapted composite index rather than a fully established stand-alone instrument. It refers to children’s preparedness to use digital resources for school-related learning. This framing is narrower than broad digital-divide and digital-competence frameworks because it focuses specifically on children’s immediate preparedness to engage with digital learning tasks. It is also distinct from digital self-efficacy: digital readiness concerns readiness-related conditions and foundational capacities, whereas digital self-efficacy concerns children’s beliefs about whether they can successfully use digital tools to complete learning tasks^10,11^.

Learning engagement is a core process variable for understanding educational quality. It reflects students’ behavioral, emotional, and cognitive investment in learning activities and is closely tied to students’ motivational beliefs, task values, achievement, persistence, and adjustment^12–16^. In digital learning contexts, engagement may also depend on learner psychology and adaptive participation. For example, recent research on AI-mediated informal digital learning has shown that engagement is intertwined with learners’ growth mindset, enjoyment, and adaptability^17^. Yet current research has paid relatively limited attention to how rural children’s digital readiness is associated with later learning engagement, especially through internal competence beliefs and family-based contextual supports.

To address this gap, the present study used two-wave time-lagged data from upper-grade primary-school students in rural Sichuan, China. We examined whether T1 digital readiness was associated with T2 learning engagement, whether T2 digital self-efficacy statistically accounted for part of this association, and whether T1 family support strengthened the first-stage association from digital readiness to digital self-efficacy. The model was tested while controlling for baseline learning engagement and key demographic covariates, which allowed the analysis to focus on subsequent engagement rather than cross-sectional differences alone. This design does not establish causality, but it provides stronger longitudinal evidence than a single-wave survey.

### Digital readiness and learning engagement

Learning engagement is generally conceptualized as students’ behavioral participation, emotional involvement, and cognitive effort in learning activities^13^. It is not merely a by-product of instruction; rather, it is a central indicator of how students participate in the learning process^14,18^. This multidimensional construct has also been widely operationalized in upper elementary and secondary education research^19^. In digital contexts, however, engagement may depend not only on pedagogical design, but also on whether students can enter technology-mediated learning spaces, understand digital tasks, use learning resources, and sustain participation when digital tools are required^20–22^.

In the present study, digital readiness goes beyond device ownership or internet access alone. It is defined as children’s preparedness to use digital resources for school-related learning and includes three theoretically informed components: learning-relevant access conditions, foundational digital capability, and practical learning preparedness. This structure is grounded in digital-divide and digital-competence perspectives. Digital-divide theory conceptualizes inequality as a layered process involving access, skills, use, and outcomes^7^, and later work has shown that when basic access expands, inequalities increasingly shift toward differences in usage quality and digital skills^8^. Digital education frameworks similarly emphasize that educational benefits depend not only on whether technology is available, but also on whether learners can use digital resources for learning purposes^1,3,23^. Accordingly, access conditions represent children’s opportunity to enter digital learning spaces, foundational digital capability reflects their ability to search for information, use learning platforms, and judge learning-related digital content, and practical learning preparedness captures whether they are ready to apply these resources to school tasks rather than to general or entertainment-oriented device use.

This structure is especially relevant for upper-grade rural primary-school students. Evidence from Chinese primary-school samples suggests that rural students may differ from urban students in information and communication technology literacy and online learning self-efficacy^6^, although such differences should be understood as contextual rather than universal. At the same time, children aged 10 to 12 are beginning to complete school-related digital tasks with some independence, but their digital learning remains tied to concrete conditions such as available devices, stable connection, basic operational skills, and the ability to use technology for educational purposes. A highly abstract digital-competence construct may therefore be difficult for this age group to report reliably, whereas the present three-component structure uses concrete, behaviorally anchored indicators that correspond to their everyday learning experience.

Digital readiness is expected to be associated with later learning engagement because engagement involves sustained behavioral participation, emotional involvement, and cognitive effort in learning activities^13,14,18^. Children with stronger readiness-related conditions may be more able to enter digital learning activities, locate useful materials, complete assigned tasks, and persist when technical or task-related difficulties arise. These experiences can reduce participation barriers and make learning activities feel more manageable and meaningful, which may support behavioral, emotional, and cognitive engagement. By contrast, children with lower digital readiness may encounter more interruptions, confusion, or frustration when digital tools are required. Building on this reasoning, the present study treats digital readiness as a readiness-related predictor of subsequent engagement rather than as a general background descriptor. Based on this reasoning, the following hypothesis is proposed:

H1: Digital readiness is positively associated with subsequent learning engagement among rural children.

### The mediating role of digital self-efficacy

Although digital readiness may be directly associated with learning engagement by reducing barriers to technology-mediated learning, this association may also operate through students’ competence beliefs. Social cognitive theory identifies self-efficacy as a key determinant of effort, persistence, and task choice^10,24^. In digital learning contexts, digital self-efficacy refers to students’ beliefs about whether they can effectively use digital tools to complete learning tasks, solve common technical problems, and adapt to technology-based learning demands^11,25^.

The distinction between digital readiness and digital self-efficacy is important. Digital readiness concerns readiness-related conditions and foundational capacities, whereas digital self-efficacy concerns children’s perceived capability to use digital tools successfully for learning. A child may have relatively favorable access conditions and basic digital capability yet still doubt his or her ability to complete digital learning tasks. Conversely, stronger readiness-related conditions may provide more opportunities to use learning platforms, search for learning materials, complete teacher-assigned digital tasks, and solve basic technical problems. These successful experiences are one of the main sources of efficacy beliefs in social cognitive theory^10^. In this sense, digital readiness and digital self-efficacy are conceptually connected but not interchangeable.

This logic suggests a readiness-belief-engagement pathway. Prior studies indicate that self-efficacy is positively associated with digital learning participation and engagement, and that digital experience or digital literacy often relates to learning outcomes partly through efficacy beliefs^21,26,27^. In the Chinese primary-school context, ICT literacy has also been linked to online learning self-efficacy^6^. Therefore, digital self-efficacy is expected to statistically account for part of the longitudinal association between digital readiness and later learning engagement. Given the two-wave design, this pathway is examined as a statistical indirect association. Based on this reasoning, the following hypothesis is proposed:

H2: Digital self-efficacy statistically accounts for part of the association between digital readiness and learning engagement among rural children.

### The moderating role of family support

Children’s learning does not occur in isolation. Family environments shape the resources, routines, encouragement, and supervision that children bring into school learning. Ecological perspectives on development suggest that child outcomes are jointly shaped by multiple proximal environments, especially home and school^28^. In technology-based learning, family support may be especially consequential because digital learning often extends into the home and requires adult guidance, time regulation, and problem solving beyond the classroom.

Recent work shows that family involvement and family support are consistently associated with children’s engagement and learning-related adjustment^29–32^. Digital parenting research similarly suggests that family mediation, supervision, and support influence children’s digital competence, digital well-being, and educational use of technology^33–36^. These findings imply that family support may matter not only as a direct contextual resource, but also as a condition that shapes whether readiness-related resources are linked to stronger competence beliefs.

The present model positions family support as a first-stage moderator for both theoretical and contextual reasons. From a social cognitive perspective, efficacy beliefs develop through mastery experiences, social persuasion, emotional reassurance, and the interpretation of difficulties^10^. In daily rural digital learning, family members can provide these conditions in observable ways: encouraging children to try digital tasks, supervising learning-related device use, helping when technical problems occur, reminding children to manage learning time, and communicating with teachers when children encounter obstacles. These forms of support may make successful digital learning experiences more frequent and less frustrating, thereby strengthening the association between digital readiness and digital self-efficacy.

At the same time, an alternative compensatory model is plausible: family support could substitute for low readiness by helping children with fewer readiness-related resources. However, following the social-cognitive logic described above and the forms of family support relevant to rural digital learning, we expected family support to function primarily as a resource-activation context that amplifies the link between readiness and efficacy beliefs. When family support is high, children may be more likely to transform readiness-related access, capability, and preparedness into confidence in digital learning. When family support is low, readiness-related conditions may be less consistently reinforced through daily support. Accordingly, the following hypothesis is proposed:

H3: Family support moderates the association between digital readiness and digital self-efficacy, such that the positive association is stronger when family support is higher.

**Fig. 1:**
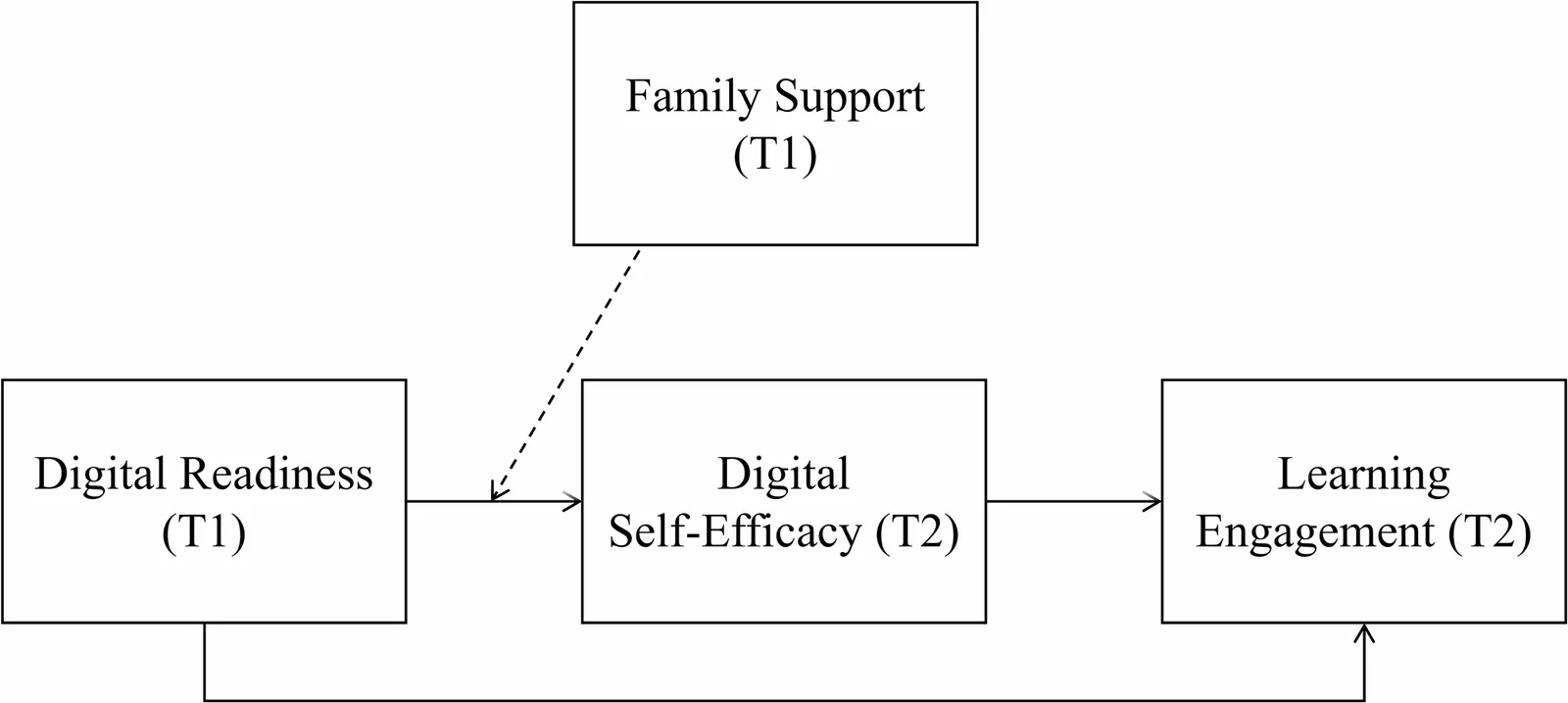
Conceptual model of the hypothesized conditional indirect association. The dashed arrow indicates the moderating role of family support. Covariates were included in the analyses but omitted from the figure for clarity.

Consequently, the indirect association between digital readiness and learning engagement through digital self-efficacy is expected to be stronger at higher levels of family support. The conceptual framework of the study is shown in Fig. 1.

## Methods

### Participants and procedure

A combination of cluster sampling and convenience sampling was used to recruit Grade 5 and Grade 6 students from 15 rural primary schools in Sichuan Province, China. The schools were accessible rural schools that agreed to participate through local educational coordination. Within participating schools, intact Grade 5 and Grade 6 classes were invited to complete the survey. This sampling approach was feasible for school-based fieldwork, but the use of accessible schools also means that the sample should not be interpreted as statistically representative of all rural Chinese primary-school students.

After school permission was obtained, school administrators and class teachers assisted with questionnaire administration. Before data collection, students and their guardians were informed about the purpose of the study, the anonymous nature of participation, and the use of the data for research only. Permission was obtained from the participants’ parents or legal guardians, and assent was obtained from the children before the survey began.

A two-wave time-lagged design was adopted. At T1, digital readiness, family support, baseline learning engagement, and demographic variables were measured. Three months later, T2 follow-up data were collected to assess digital self-efficacy and learning engagement. Separating the predictor and moderator from the follow-up mediator and outcome helped reduce purely cross-sectional interpretation, although the two-wave design cannot fully establish causal ordering between digital self-efficacy and learning engagement because both were assessed at T2.

At T1, 980 questionnaires were distributed and 896 valid responses were obtained, yielding an effective response rate of 91.43%. At T2, 789 questionnaires were returned. After anonymous code matching and data screening, 768 valid cases were successfully matched across both waves, representing 85.71% of the valid T1 sample. The final analytic sample therefore consisted of 768 students who completed both waves. Of these, 409 were in Grade 5 (53.26%) and 359 were in Grade 6 (46.74%); 404 were boys (52.60%) and 364 were girls (47.40%). The study protocol was approved by the Ethics Committee of Leshan Normal University. All questionnaires were anonymous and no real names were recorded.

### Measures

All instruments were adapted from established measures or mature measurement frameworks. Because the participants were upper-grade primary-school children living in rural areas, items were revised to ensure age-appropriate wording, contextual fit, and relevance to digital learning. The draft instrument set was reviewed by specialists in educational psychology, curriculum and instruction, and educational measurement. A pilot study was then conducted with approximately 50 rural Grade 5 and Grade 6 students who were similar to the formal participants. The pilot study focused on item clarity, reading comprehension, response time, and whether students could distinguish the meanings of readiness, confidence, support, and engagement items. Students were invited to indicate words or statements they found difficult to understand, and minor wording revisions were made before formal data collection. Reliability and validity evidence reported below was recalculated using the matched analytic sample (*N* = 768).

#### Digital readiness (T1)

Digital readiness assessed children’s readiness for participating in digital learning. Because no single established instrument fully captured this construct for rural primary-school students, we used a study-specific adapted composite index. The final 10 items were adapted from the OECD ICT Questionnaire for PISA 2022^2^ and the Digital Intelligence Quotient Scale for Chinese primary-school students^37^. The retained items represented three theoretically informed components: learning-relevant access conditions (items 1–2), foundational digital capability (items 3–7), and practical learning preparedness (items 8–10). Items primarily reflecting confidence in performing digital learning tasks were not included, to preserve conceptual distinctiveness from digital self-efficacy. Responses were rated on a 5-point Likert scale (1 = strongly disagree to 5 = strongly agree). Higher scores indicated higher digital readiness. In the matched analytic sample, Cronbach’s *α* was 0.812. The one-factor, three-component correlated-factor, and higher-order models all showed acceptable fit, supporting the use of digital readiness as a composite readiness index (see Supplementary Table S2).

The three-component structure was chosen because it reflects the conditions that jointly determine whether rural upper-primary children can engage in school-related digital learning in daily educational contexts. For this population, learning-relevant access conditions are important because home connectivity and usable devices may vary across families; foundational digital capability is also necessary because children at this age are still developing basic skills for searching, judging, and using digital information; and practical learning preparedness helps distinguish readiness for school-related digital learning from general device exposure or entertainment-oriented use. Together, these three concrete and behaviorally anchored components capture digital readiness at a level appropriate for 10- to 12-year-old children, for whom highly abstract or broad digital-competence constructs may be more difficult to report reliably. Because the three components were highly correlated and the one-factor, three-component correlated-factor, and higher-order models all showed acceptable and comparable fit (Supplementary Table S2), a parsimonious composite readiness index was retained as the operationalization that best represented children’s overall preparedness for digital learning in this population.

#### Family support (T1)

Family support assessed the emotional, instrumental, and supervisory support children perceived from family members during digital learning. The final 6-item scale was adapted from the family support dimension of the Multidimensional Scale of Perceived Social Support (MSPSS^38^; to fit the digital learning context. The revised items focused on whether family members encouraged, helped, supervised, and supported children when they used digital devices for learning. Responses were recorded on a 5-point Likert scale, with higher scores indicating stronger perceived family support. Cronbach’s *α* was 0.766 in the matched analytic sample.

#### Learning engagement (T1 and T2)

Learning engagement was measured at both waves to capture baseline and follow-up engagement. The final 9-item scale covered behavioral, emotional, and cognitive engagement and drew on the three-dimensional framework proposed by Fredricks et al.^13^, with adaptation based on upper-elementary engagement measures described by Fredricks et al.^19^,. T1 and T2 used the same items and the same 5-point Likert response format. Higher scores reflected higher engagement. Cronbach’s *α* values were 0.799 at T1 and 0.858 at T2. Because the same engagement scale was used across waves, longitudinal measurement invariance was examined; partial metric and partial scalar invariance were supported (see Supplementary Table S4).

#### Digital self-efficacy (T2)

Digital self-efficacy assessed students’ confidence in using digital tools to complete teacher-assigned learning tasks, search for learning materials, use learning platforms, and handle basic technical difficulties. The final 6-item scale was adapted from the Online Learning Self-Efficacy Scale^25^ for younger students and digital learning in primary education. Responses were rated on a 5-point Likert scale, with higher scores indicating stronger digital self-efficacy. Cronbach’s *α* was 0.724 in the matched analytic sample, indicating acceptable internal consistency for an adapted child-report scale.

### Statistical analysis

All analyses were conducted on the matched analytic sample. Descriptive statistics, Pearson correlations, reliability coefficients, confirmatory factor analyses (CFAs), and measurement validation indices were first computed. CFA model fit was evaluated using the comparative fit index (CFI), Tucker-Lewis index (TLI), root mean square error of approximation (RMSEA), and standardized root mean square residual (SRMR)^39^. For the T1–T2 learning engagement scale, longitudinal measurement invariance was assessed by comparing configural, metric, and scalar models; changes in CFI, RMSEA, and SRMR were considered when evaluating invariance^40,41^.

Common method bias was assessed in two ways. Harman’s single-factor test was retained only as a preliminary diagnostic, as this approach alone is insufficient for ruling out common method bias^42^. In addition, a one-factor CFA model was compared with the five-factor measurement model to evaluate whether the covariance among the focal self-report items could be better explained by a dominant single-factor structure. A marker-variable approach was not used because the study did not include a theoretically unrelated marker variable. Accordingly, common method bias could not be fully ruled out and was addressed again in the limitations.

The main model entered T1 digital readiness as the predictor, T2 learning engagement as the outcome, T2 digital self-efficacy as the mediator, and T1 family support as the moderator. Gender, grade, device availability, internet quality, parental education, digital learning duration, and baseline learning engagement at T1 were controlled. Gender was coded as 1 = male and 2 = female. Grade was coded as 1 = Grade 5 and 2 = Grade 6. PROCESS Model 7^43^ was used to test the first-stage moderated indirect association. This model specifies moderation on the first-stage path, and this specification was theoretically grounded in social cognitive and ecological perspectives. Bias-corrected bootstrap confidence intervals were estimated from 5,000 resamples. Continuous variables were mean-centered before the interaction term was created. Both unstandardized coefficients (*B*) and standardized coefficients (*β*) are reported.

Because students were nested within 15 rural primary schools, intraclass correlations (ICCs) were calculated for T2 digital self-efficacy and T2 learning engagement. Robustness checks were then conducted using school-clustered standard errors. These analyses were used to evaluate whether the main findings were robust to potential school-level clustering.

## Results

### Measurement validation and common method diagnostics

Table 1 summarizes the main reliability and validity evidence based on the matched analytic sample. Cronbach’s *α* values ranged from 0.724 to 0.858, and composite reliability (CR) values ranged from 0.724 to 0.858. Standardized factor loadings were positive and ranged from 0.475 to 0.690. Although the AVE values were below the conventional 0.50 threshold, convergent validity can still be considered acceptable when composite reliability exceeds 0.60^44^, which was the case for all constructs. Lower AVE values are not unexpected for adapted, broad-content child-report scales that retain heterogeneous item content to preserve construct coverage. Discriminant validity was supported by two criteria. First, for every pair of conceptually distinct constructs, the square root of the AVE exceeded the corresponding inter-construct correlation. For example, the square root of AVE for digital readiness was 0.549, which was higher than its correlation with digital self-efficacy (*r* = 0.341). The T1-T2 learning engagement pair was not treated as evidence against discriminant validity because it reflects the same construct measured across waves rather than two distinct constructs. Second, all HTMT values were below 0.85 (Supplementary Table S3). Detailed item-level loadings, corrected item-total correlations, alternative digital readiness factor models, discriminant validity results, and measurement invariance results are provided in Supplementary Tables S1-S4.

**Table 1:** Reliability and measurement validation summary.

Construct	Items	α	CR	AVE	Loading range	CFI	TLI	RMSEA	SRMR
Digital readiness (T1)	10	0.812	0.812	0.301	0.528–0.576	0.996	0.995	0.014	0.024
Family support (T1)	6	0.766	0.766	0.354	0.568–0.626	0.999	0.998	0.011	0.019
Learning engagement (T1)	9	0.799	0.800	0.308	0.491–0.583	1.000	1.000	0.000	0.022
Digital self-efficacy (T2)	6	0.724	0.724	0.306	0.475–0.606	1.000	1.000	0.000	0.017
Learning engagement (T2)	9	0.858	0.858	0.402	0.574–0.690	0.996	0.995	0.020	0.022

*α* = Cronbach’s alpha; CR = composite reliability; AVE = average variance extracted. All results were based on the matched analytic sample (*N* = 768). Detailed item-level results are reported in the Supplementary Materials. CR was computed from standardized factor loadings; because item loadings within each construct were similar in magnitude, CR values were very close to Cronbach’s *α*.

Harman’s single-factor test extracted 5 factors with eigenvalues greater than 1, and the first factor accounted for 19.24% of the variance. This result did not indicate a dominant single factor. However, because this test is limited, an additional CFA comparison was conducted. The one-factor CFA model fit the data poorly (CFI = 0.607, TLI = 0.586, RMSEA = 0.070, SRMR = 0.089), whereas the five-factor measurement model fit well (CFI = 0.990, TLI = 0.989, RMSEA = 0.011, SRMR = 0.030). These results did not support a dominant single-factor explanation, although common-method concerns cannot be completely excluded because all focal variables were measured through student self-reports. These additional common method diagnostics are summarized in Supplementary Table S5.

### Attrition analysis

To examine whether sample attrition introduced systematic bias, students who completed both waves (retained group, *n* = 768) were compared with those who completed only T1 (attrited group, *n* = 128) on demographic characteristics and T1 core variables. Welch’s independent-samples *t* tests were used for continuous and ordinal variables, and a chi-square test was used for gender. To avoid relying only on non-significant tests, effect sizes were also reported (Table 2).

**Table 2:** Baseline differences between retained and attrited participants.

Variable	Retained M (SD)/%	Attrited M (SD)/%	t/χ²	*p*	Effect size
Grade	1.47 (0.50)	1.48 (0.50)	*t* = −0.35	0.724	*d* = −0.034
Device availability	3.39 (1.13)	3.47 (1.19)	*t* = −0.67	0.504	*d* = −0.066
Internet quality	3.52 (1.14)	3.63 (1.14)	*t* = −1.08	0.282	*d* = −0.103
Parental education	2.72 (1.15)	2.68 (1.20)	*t* = 0.32	0.749	*d* = 0.032
Digital learning duration	3.00 (1.10)	3.09 (0.98)	*t* = −0.97	0.335	*d* = −0.085
Digital readiness (T1)	3.27 (0.64)	3.30 (0.69)	*t* = −0.49	0.624	*d* = −0.050
Family support (T1)	3.39 (0.66)	3.44 (0.66)	*t* = −0.66	0.511	*d* = −0.063
Learning engagement (T1)	3.56 (0.59)	3.60 (0.56)	*t* = −0.61	0.543	*d* = −0.056
Gender (male %)	52.6%	53.9%	*χ²*(1) = 0.03	0.859	*V* = 0.006

Retained group *n* = 768; attrited group *n* = 128. Gender was coded as 1 = male and 2 = female; grade was coded as 1 = Grade 5 and 2 = Grade 6. *d* = Cohen’s *d*; *V* = Cramé*r*’s *V*.

The results showed no significant between-group differences for grade, device availability, internet quality, parental education, digital learning duration, digital readiness, family support, or T1 learning engagement. Gender distribution also did not differ significantly. The effect sizes were very small across comparisons, suggesting no clear selective attrition pattern.

### Descriptive statistics and correlations

Descriptive statistics and bivariate correlations are presented in Table 3. The strongest correlation was between T1 and T2 learning engagement, indicating stability in the repeated engagement measure. Digital readiness was moderately correlated with digital self-efficacy and T2 learning engagement, but its correlation with digital self-efficacy was not high enough to suggest redundancy.

**Table 3:** Descriptive statistics and correlations among the variables (*N* = 768).

Variable	M	SD	1	2	3	4	5	6	7	8	9	10	11
1. Gender	1.474	0.500	1										
2. Grade	1.467	0.499	−0.006	1									
3. Device availability	3.393	1.130	0.016	0.018	1								
4. Internet quality	3.516	1.143	−0.020	−0.055	−0.040	1							
5. Parental education	2.716	1.150	−0.063	−0.034	−0.034	−0.012	1						
6. Digital learning duration	3.001	1.099	−0.020	0.054	0.005	−0.047	0.010	1					
7. Digital readiness (T1)	3.269	0.636	0.020	0.051	0.337***	0.231***	0.161***	0.152***	1				
8. Family support (T1)	3.395	0.657	−0.032	0.026	0.133***	0.100**	0.155***	0.006	0.099**	1			
9. Learning engagement (T1)	3.564	0.595	−0.040	0.135***	0.162***	0.002	0.055	0.001	0.185***	0.120***	1		
10. Digital self-efficacy (T2)	3.410	0.569	0.056	0.065	0.128***	0.080*	0.146***	0.064	0.341***	0.166***	0.209***	1	
11. Learning engagement (T2)	3.454	0.724	−0.006	0.079*	0.193***	0.040	0.102**	0.049	0.335***	0.158***	0.644***	0.393***	1

1 = gender; 2 = grade; 3 = device availability; 4 = internet quality; 5 = parental education; 6 = digital learning duration; 7 = digital readiness; 8 = family support; 9 = learning engagement (T1); 10 = digital self-efficacy; 11 = learning engagement (T2). * *p* < 0.05. ** *p* < 0.01. *** *p* < 0.001.

### Mediation and moderated mediation analyses

Regression analyses and bias-corrected bootstrap tests were used to evaluate the hypothesized model. All models controlled for gender, grade, device availability, internet quality, parental education, digital learning duration, and baseline learning engagement at T1.

In the total-effect model, digital readiness was positively associated with T2 learning engagement (*B* = 0.237, *SE* = 0.035, *β* = 0.208, *t* = 6.81, *p* < 0.001). In the mediation model, digital readiness was positively associated with T2 digital self-efficacy (*B* = 0.253, *SE* = 0.034, *β* = 0.283, *t* = 7.34, *p* < 0.001), and T2 digital self-efficacy was positively associated with T2 learning engagement (*B* = 0.282, *SE* = 0.035, *β* = 0.221, *t* = 8.00, *p* < 0.001). The direct association between digital readiness and T2 learning engagement remained significant after the mediator was entered (*B* = 0.166, *SE* = 0.035, *β* = 0.146, *t* = 4.79, *p* < 0.001). The bootstrap indirect association was 0.071, with a 95% confidence interval of [0.047, 0.100], indicating a significant partial indirect association (Table 4).

**Table 4:** Regression results for total-effect and mediation models.

Model	Predictor	B	SE	β	t	95% CI	*p*	*R*²	F
Total-effect model: Learning engagement (T2)	Gender	0.023	0.039	0.016	0.59	[−0.053, 0.098]	0.558	0.465	82.60
	Grade	−0.019	0.039	−0.013	−0.49	[−0.096, 0.058]	0.625		
	Device availability	0.017	0.018	0.027	0.93	[−0.019, 0.053]	0.355		
	Internet quality	−0.004	0.018	−0.007	−0.25	[−0.039, 0.030]	0.803		
	Parental education	0.023	0.017	0.036	1.32	[−0.011, 0.056]	0.188		
	Digital learning duration	0.011	0.018	0.017	0.63	[−0.024, 0.046]	0.530		
	Digital readiness (T1)	0.237	0.035	0.208	6.81	[0.169, 0.306]	< 0.001		
	Learning engagement (T1)	0.732	0.033	0.602	21.90	[0.667, 0.798]	< 0.001		
Mediator model: Digital self-efficacy (T2)	Gender	0.072	0.038	0.064	1.89	[−0.003, 0.147]	0.059	0.152	16.95
	Grade	0.039	0.039	0.034	1.01	[−0.037, 0.115]	0.312		
	Device availability	0.006	0.018	0.012	0.32	[−0.030, 0.042]	0.748		
	Internet quality	0.010	0.017	0.020	0.58	[−0.024, 0.044]	0.565		
	Parental education	0.048	0.017	0.098	2.86	[0.015, 0.082]	0.004		
	Digital learning duration	0.011	0.018	0.021	0.60	[−0.024, 0.045]	0.546		
	Digital readiness (T1)	0.253	0.034	0.283	7.34	[0.186, 0.321]	< 0.001		
	Learning engagement (T1)	0.140	0.033	0.147	4.24	[0.075, 0.205]	< 0.001		
Direct-effect model: Learning engagement (T2)	Gender	0.002	0.037	0.002	0.06	[−0.071, 0.075]	0.952	0.507	86.62
	Grade	−0.030	0.038	−0.021	−0.80	[−0.104, 0.044]	0.423		
	Device availability	0.015	0.018	0.024	0.87	[−0.019, 0.050]	0.385		
	Internet quality	−0.007	0.017	−0.011	−0.43	[−0.040, 0.026]	0.669		
	Parental education	0.009	0.017	0.014	0.54	[−0.024, 0.041]	0.590		
	Digital learning duration	0.008	0.017	0.012	0.48	[−0.025, 0.042]	0.632		
	Digital readiness (T1)	0.166	0.035	0.146	4.79	[0.098, 0.234]	< 0.001		
	Digital self-efficacy (T2)	0.282	0.035	0.221	8.00	[0.213, 0.351]	< 0.001		
	Learning engagement (T1)	0.693	0.033	0.569	21.31	[0.629, 0.757]	< 0.001		

*B* = unstandardized coefficient; *β* = standardized coefficient. Covariates listed in Sect. 2.3 were controlled. *R*² and *F* are model-level statistics and are displayed in the first row of each model block; all model-level *F* tests were significant at *p* < 0.001.

**Fig. 2:**
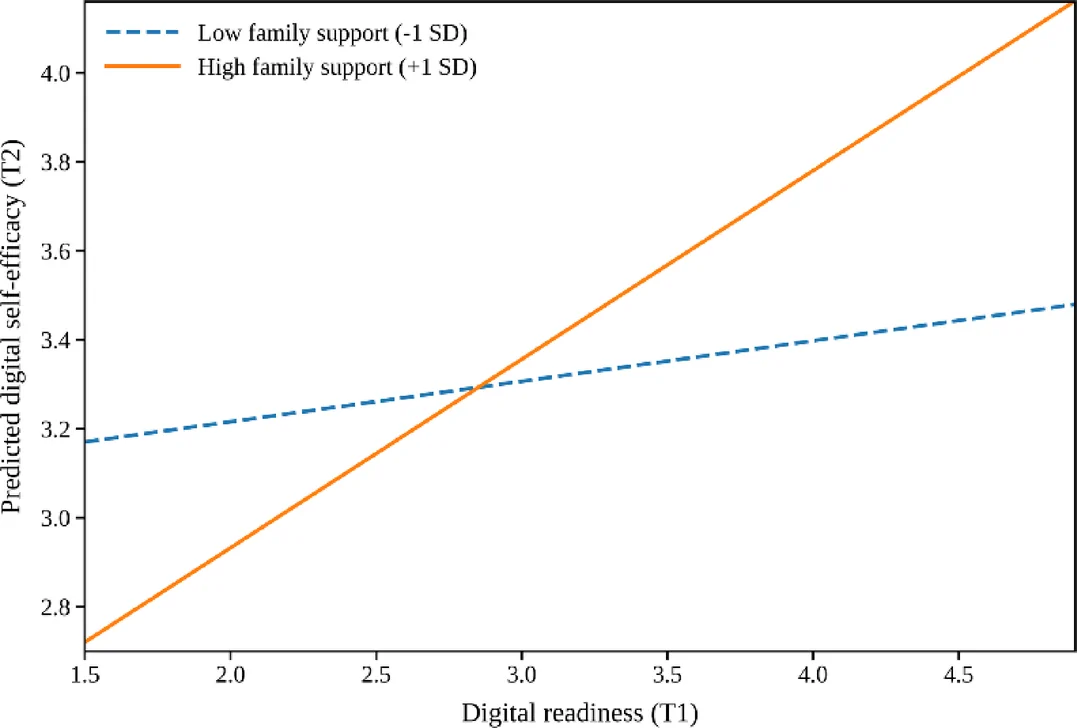
Simple slopes for the association between digital readiness and T2 digital self-efficacy at low and high levels of family support.

In the first-stage moderated model, family support was positively associated with digital self-efficacy (*B* = 0.106, *SE* = 0.029, *β* = 0.123, *t* = 3.64, *p* < 0.001). The interaction between digital readiness and family support was also significant (*B* = 0.253, *SE* = 0.043, *β* = 0.191, *t* = 5.85, *p* < 0.001), indicating that the readiness-efficacy association varied by family support. The full first-stage moderated model is reported in Supplementary Table S6. Table 5 summarizes the key simple slopes and conditional indirect associations, and the full conditional results are provided in Supplementary Table S7 (Fig. 2).

**Table 5:** Simple slopes and conditional indirect associations.

Effect	Estimate	SE/Boot SE	t	95% CI	*p*
Simple slope at low family support (−1 *SD*)	0.091	0.044	2.10	[0.006, 0.177]	0.036
Simple slope at high family support (+ 1 *SD*)	0.424	0.044	9.57	[0.337, 0.511]	< 0.001
Conditional indirect association at low family support	0.026	0.013	–	[0.001, 0.053]	–
Conditional indirect association at mean family support	0.073	0.013	–	[0.048, 0.100]	–
Conditional indirect association at high family support	0.119	0.019	–	[0.084, 0.158]	–
Index of conditional indirect association	0.071	0.014	–	[0.045, 0.101]	–

Family support was mean-centered. Bootstrap CIs were based on 5,000 resamples. *p* values are reported for simple slopes only; conditional indirect associations and the index were evaluated by bootstrap 95% CIs.

### School-clustered robustness checks

Because students were sampled from 15 schools, potential school-level clustering was examined. The ICC was 0.000 for T2 digital self-efficacy and 0.004 for T2 learning engagement, indicating very small between-school variance in these outcomes. The key findings remained significant when school-clustered standard errors were used. For example, the association between digital readiness and T2 learning engagement remained significant in the total-effect model (*B* = 0.237, cluster-robust *SE* = 0.040, *p* < 0.001), and the interaction between digital readiness and family support remained significant in the mediator model (*B* = 0.253, cluster-robust *SE* = 0.036, *p* < 0.001). Because the number of school clusters was limited, these cluster-robust analyses should be interpreted as robustness checks rather than definitive multilevel evidence; however, the near-zero ICCs suggested that school-level dependence had little influence on the estimates. Full robustness results are reported in Supplementary Table S8.

## Discussion

Using two-wave time-lagged data from upper-grade primary-school students in rural China, this study examined how digital readiness was longitudinally associated with later learning engagement and whether digital self-efficacy and family support were involved in this association. The main findings were consistent with the hypothesized model: T1 digital readiness was associated with T2 learning engagement after baseline engagement and demographic covariates were controlled; digital self-efficacy partially accounted for this association; and family support strengthened the readiness-efficacy association. Accordingly, the findings are discussed as longitudinal associations rather than as evidence of causal mechanisms.

### From access-based digital inequality to readiness-based digital inequality

The first contribution of this study is to shift attention from access-based digital inequality to readiness-based digital inequality in rural primary education. Prior digital-divide frameworks emphasize that inequalities move from material access to skills, use, and outcomes^7,8^. The present findings extend this logic to rural children’s learning engagement by showing that digital readiness is associated with later engagement even when baseline engagement is controlled. This does not mean that device access is unimportant. Rather, it suggests that educational benefits depend on whether children can turn access into learning-relevant participation.

This point is practically important in rural education. A school may provide digital resources, but children still differ in whether they can connect to learning tasks, locate useful materials, use learning platforms, and persist when difficulties occur. Digital readiness captures this proximal learning preparedness. Thus, the study’s novelty lies not simply in applying a moderated mediation model to a rural Chinese sample, but in specifying a readiness-based pathway through which digital inequality may be linked to subsequent engagement.

### Digital self-efficacy as a partial pathway

The second contribution concerns digital self-efficacy. Consistent with social cognitive theory, readiness-related conditions were associated with stronger competence beliefs, and these beliefs were in turn associated with later engagement^10,24^. This finding is consistent with studies linking digital literacy, digital attitudes, and digital self-efficacy to online learning engagement^17,21,26^. At the same time, the mediation was partial. The indirect association was approximately 30% of the total association, meaning that a substantial proportion of the readiness-engagement association may operate through other pathways.

Because student engagement is shaped by multiple motivational, cognitive, and contextual conditions^12,14,16,18^, several alternative mechanisms should be considered. Digital readiness may reduce task barriers, increase access to learning materials, improve the efficiency of completing assignments, or create more opportunities for successful participation. It may also be associated with prior achievement, general cognitive ability, teacher digital support, school digital climate, or parental digital literacy, none of which was directly measured in this study. The present findings therefore support digital self-efficacy as one relevant psychological pathway, but they do not imply that it is the only or dominant pathway.

### Family support as a resource-activation context

The third contribution concerns the contextual role of family support. The results were more consistent with an amplification or resource-activation pattern than with a compensatory pattern: the association between digital readiness and digital self-efficacy was substantially stronger at high family support than at low family support. In educational terms, this pattern suggests that readiness-related resources are more likely to be reflected in children’s confidence when families provide concrete supports such as encouragement, supervision, troubleshooting, and learning-time regulation. This interpretation is especially relevant in rural digital learning contexts, where digital learning may require children to coordinate school tasks, home devices, and family guidance in everyday routines.

This interpretation connects ecological and social cognitive perspectives. Ecological theory highlights the importance of proximal family contexts, whereas social cognitive theory explains why encouragement, social persuasion, guided mastery experiences, and emotional reassurance can strengthen efficacy beliefs^10,28^. In rural digital learning, family members may support children in concrete ways: reminding them to use devices for learning rather than entertainment, helping them solve simple technical problems, encouraging them when online tasks are difficult, and contacting teachers when problems exceed the family’s capacity. These forms of support may make children’s readiness-related resources more likely to become stable confidence in digital learning.

The compensatory possibility should not be dismissed. In some contexts, strong family support may substitute for low digital readiness by helping children overcome limited access or skills. Future studies should test whether amplification and compensation operate differently across subgroups, such as children with very low access, different levels of parental digital literacy, or different school digital-support conditions. The present findings suggest that in this sample, however, family support functioned primarily as a context that strengthened the readiness-efficacy link.

### Practical implications

The findings suggest several context-grounded implications for rural digital education. First, rural schools may need to avoid evaluating digital learning only by device provision or platform adoption. They may also consider whether children can use digital tools to find learning materials, complete teacher-assigned tasks, and handle common difficulties. Second, family guidance should be designed around low-threshold, observable forms of support. Many rural families may not be able to provide advanced technical instruction, but they can encourage children, supervise learning-related device use, help children organize time, and communicate with teachers when problems arise. Third, schools can provide short parent guidance cards, class-level communication routines, or simple troubleshooting channels so that families know how to support children’s digital learning without excessive burden.

### Limitations and future directions

Several limitations should be acknowledged. First, although the two-wave time-lagged design improves on a single-wave survey, it does not establish strict causality. Digital self-efficacy and learning engagement were both measured at T2, so the temporal ordering between the mediator and outcome cannot be fully established. Future studies should use three-wave or multi-wave designs to separate predictor, mediator, and outcome over time.

Second, all focal variables were measured through student self-reports. The participants were 10- to 12-year-old children, and their responses may be affected by reading comprehension, response consistency, and social desirability. The pilot study and age-appropriate wording helped reduce this concern, but future research should incorporate teacher ratings, parent reports, learning-platform records, classroom observations, or behavioral engagement indicators. Relatedly, although additional diagnostics did not support a dominant single-factor explanation, common method bias cannot be fully ruled out because all core variables came from the same respondent source and no theoretically unrelated marker variable was included.

Third, the adapted measures showed acceptable composite reliability but relatively modest AVE values, indicating acceptable but not strong convergent validity. Future studies should further refine these child-report scales and examine their measurement properties in other rural child samples.

Fourth, although the ICCs were small and school-clustered standard errors supported the robustness of the findings, the number of school clusters was limited. In addition, the sample was drawn from Grade 5 and Grade 6 students in rural Sichuan. The findings are contextually informative but should be generalized cautiously to other regions, age groups, and urban-rural contexts.

Fifth, the study did not include prior academic achievement, general cognitive ability, objective digital-use data, detailed parental digital literacy, or school-level variables such as school digital infrastructure, teacher digital support, and school digital climate. These omitted variables may partly explain the associations observed here and should be incorporated in future research.

## Conclusion

Based on two-wave time-lagged data from rural Chinese primary-school students, this study found that digital readiness was associated with later learning engagement both directly and via digital self-efficacy. Family support strengthened the association between digital readiness and digital self-efficacy, thereby strengthening the conditional indirect association with engagement. These findings suggest that rural children’s learning engagement in digital contexts is linked not only to access, but also to their readiness to learn with technology, competence beliefs, and concrete family support. The findings should be interpreted as longitudinal associations rather than causal effects.

## Supplementary Information

Below is the link to the electronic supplementary material.


Supplementary Material 1


## Data Availability

Because the dataset involves minors, the de-identified data are not publicly posted. The de-identified data supporting the findings of this study are available from the corresponding author upon reasonable request and subject to approval by the relevant ethics committee and school data-protection requirements.
