# Supplementary material for: Digital readiness and family support are associated with later learning engagement through digital self-efficacy among rural Chinese children
Source: Sci Rep. 2026 Jun 14;16:27185. doi: 10.1038/s41598-026-58145-2 (PMC13526775; doi:10.1038/s41598-026-58145-2)
Supplement: Supplementary file 1 [file 41598_2026_58145_MOESM1_ESM.docx]

**Supplementary Materials**

Digital readiness and family support are associated with later learning engagement through digital self-efficacy among rural Chinese children

This supplementary file reports detailed measurement validation, first-stage moderation, full conditional indirect association, and school-clustered robustness results for the revised manuscript.

**Table S1.** *Item-level descriptive statistics, standardized loadings, and reliability diagnostics.*

| **Construct** | **Item** | ***M*** | ***SD*** | **Std. loading** | **Corrected item-total *r*** | ***α* if item deleted** |
| --- | --- | --- | --- | --- | --- | --- |
| Digital readiness (T1) | DR01 | 3.180 | 1.063 | 0.553 | 0.493 | 0.794 |
|  | DR02 | 3.555 | 1.035 | 0.528 | 0.471 | 0.797 |
|  | DR03 | 3.151 | 1.065 | 0.553 | 0.494 | 0.794 |
|  | DR04 | 3.348 | 1.042 | 0.552 | 0.492 | 0.795 |
|  | DR05 | 3.320 | 1.025 | 0.530 | 0.471 | 0.797 |
|  | DR06 | 3.246 | 1.046 | 0.576 | 0.513 | 0.792 |
|  | DR07 | 3.042 | 1.051 | 0.529 | 0.472 | 0.797 |
|  | DR08 | 3.189 | 1.077 | 0.565 | 0.503 | 0.793 |
|  | DR09 | 3.251 | 1.019 | 0.545 | 0.487 | 0.795 |
|  | DR10 | 3.408 | 1.016 | 0.557 | 0.496 | 0.794 |
| Family support (T1) | FS01 | 3.323 | 0.981 | 0.595 | 0.508 | 0.732 |
|  | FS02 | 3.490 | 0.973 | 0.568 | 0.488 | 0.737 |
|  | FS03 | 3.318 | 0.975 | 0.586 | 0.502 | 0.734 |
|  | FS04 | 3.371 | 0.966 | 0.606 | 0.517 | 0.730 |
|  | FS05 | 3.497 | 0.946 | 0.586 | 0.503 | 0.733 |
|  | FS06 | 3.371 | 0.968 | 0.626 | 0.533 | 0.726 |
| Learning engagement (T1) | LE01_T1 | 3.514 | 0.971 | 0.578 | 0.511 | 0.777 |
|  | LE02_T1 | 3.566 | 0.972 | 0.578 | 0.508 | 0.777 |
|  | LE03_T1 | 3.486 | 0.991 | 0.583 | 0.515 | 0.776 |
|  | LE04_T1 | 3.521 | 0.948 | 0.581 | 0.512 | 0.777 |
|  | LE05_T1 | 3.529 | 0.972 | 0.533 | 0.473 | 0.782 |
|  | LE06_T1 | 3.612 | 0.907 | 0.536 | 0.474 | 0.782 |
|  | LE07_T1 | 3.637 | 0.941 | 0.553 | 0.487 | 0.780 |
|  | LE08_T1 | 3.573 | 0.993 | 0.554 | 0.488 | 0.780 |
|  | LE09_T1 | 3.641 | 0.942 | 0.491 | 0.436 | 0.787 |
| Digital self-efficacy (T2) | D*SE*01_T2 | 3.443 | 0.926 | 0.606 | 0.497 | 0.673 |
|  | D*SE*02_T2 | 3.419 | 0.854 | 0.526 | 0.436 | 0.692 |
|  | D*SE*03_T2 | 3.525 | 0.860 | 0.557 | 0.464 | 0.684 |
|  | D*SE*04_T2 | 3.405 | 0.869 | 0.596 | 0.488 | 0.676 |
|  | D*SE*05_T2 | 3.349 | 0.859 | 0.475 | 0.398 | 0.702 |
|  | D*SE*06_T2 | 3.322 | 0.899 | 0.548 | 0.456 | 0.686 |
| Learning engagement (T2) | LE01_T2 | 3.589 | 1.077 | 0.690 | 0.630 | 0.838 |
|  | LE02_T2 | 3.400 | 1.029 | 0.611 | 0.565 | 0.844 |
|  | LE03_T2 | 3.643 | 1.032 | 0.574 | 0.529 | 0.848 |
|  | LE04_T2 | 3.335 | 1.090 | 0.658 | 0.603 | 0.841 |
|  | LE05_T2 | 3.523 | 1.062 | 0.658 | 0.603 | 0.841 |
|  | LE06_T2 | 3.523 | 1.041 | 0.639 | 0.584 | 0.843 |
|  | LE07_T2 | 3.486 | 1.044 | 0.616 | 0.569 | 0.844 |
|  | LE08_T2 | 3.378 | 1.111 | 0.620 | 0.571 | 0.844 |
|  | LE09_T2 | 3.211 | 1.043 | 0.635 | 0.581 | 0.843 |

*Note.* Std. loading = standardized loading from the one-factor CFA model for each construct. Corrected item-total *r* was computed with the item excluded from the total score.

**Table S2.** *Alternative measurement models for digital readiness.*

| **Model** | ***χ²*** | ***df*** | **CFI** | **TLI** | **RMSEA** | **SRMR** |
| --- | --- | --- | --- | --- | --- | --- |
| One-factor composite model | 40.27 | 35 | 0.996 | 0.995 | 0.014 | 0.024 |
| Three correlated components | 35.96 | 32 | 0.997 | 0.996 | 0.013 | 0.022 |
| Higher-order readiness model | 36.18 | 32 | 0.997 | 0.996 | 0.013 | 0.022 |

*Note.* The three theoretically informed components were learning-relevant access conditions (DR01-DR02), foundational digital capability (DR03-DR07), and practical learning preparedness (DR08-DR10). The high correlations among the three components supported using a composite readiness index.

**Table S3.** *Discriminant validity among focal constructs.*

| **Construct** | **Digital readiness** | **Family support** | **Learning engagement (T1)** | **Digital self-efficacy** | **Learning engagement (T2)** |
| --- | --- | --- | --- | --- | --- |
| Digital readiness (T1) | — | 0.099 | 0.185 | 0.341 | 0.335 |
| Family support (T1) | 0.135 | — | 0.120 | 0.166 | 0.158 |
| Learning engagement (T1) | 0.231 | 0.161 | — | 0.209 | 0.644 |
| Digital self-efficacy (T2) | 0.446 | 0.223 | 0.273 | — | 0.393 |
| Learning engagement (T2) | 0.401 | 0.195 | 0.778 | 0.499 | — |

*Note.* Values above the diagonal are latent/scale correlations; values below the diagonal are HTMT values. Digital readiness and digital self-efficacy were moderately correlated (*r* = 0.341), and their HTMT value was 0.446. Learning engagement is reported separately for T1 and T2 to distinguish the baseline and follow-up measurements of the same construct.

**Table S4.** *Longitudinal measurement invariance of learning engagement across T1 and T2.*

| **Model** | ***χ²*** | ***df*** | **CFI** | **TLI** | **RMSEA** | **SRMR** | **ΔCFI** | **ΔRMSEA** | **ΔSRMR** |
| --- | --- | --- | --- | --- | --- | --- | --- | --- | --- |
| Configural | 145.49 | 133 | 0.997 | 0.996 | 0.011 | 0.026 | — | — | — |
| Full metric | 206.10 | 142 | 0.983 | 0.982 | 0.024 | 0.047 | -0.014 | 0.013 | 0.021 |
| Partial metric | 172.83 | 138 | 0.991 | 0.990 | 0.018 | 0.036 | -0.006 | 0.007 | 0.010 |
| Partial scalar | 213.17 | 141 | 0.981 | 0.979 | 0.026 | 0.036 | -0.010 | 0.008 | 0.000 |

*Note.* Partial metric invariance freed the loadings of LE01, LE04, LE05, and LE09. Partial scalar invariance additionally freed the intercepts of LE01, LE02, LE04, LE05, LE06, and LE09. These results support cautious use of T1 engagement as a baseline control while acknowledging that full invariance was not obtained.

**Table S5.** *Additional common method bias diagnostics.*

| **Test** | **Index** | **Result** | **Interpretation** |
| --- | --- | --- | --- |
| Harman single-factor diagnostic | First factor variance | 19.24% | Five factors had eigenvalues greater than 1 |
| One-factor CFA model | CFI/TLI/RM*SE*A/SRMR | 0.607/0.586/0.070/0.089 | Poor fit |
| Five-factor measurement model | CFI/TLI/RM*SE*A/SRMR | 0.990/0.989/0.011/0.030 | Good fit |

*Note.* These diagnostics do not eliminate common-method concerns, but they do not support a dominant single-factor explanation. A marker-variable approach was not used because no theoretically unrelated marker variable was included in the study design.

**Table S6.** *First-stage moderated model predicting digital self-efficacy (T2).*

| **Dependent variable** | **Predictor** | ***B*** | ***SE*** | ***β*** | ***t*** | **95% CI** | ***p*** | ***R²*** | ***F*** |
| --- | --- | --- | --- | --- | --- | --- | --- | --- | --- |
| Digital self-efficacy (T2) | Gender | 0.081 | 0.037 | 0.071 | 2.17 | [0.008, 0.154] | 0.031 | 0.199 | 18.80 |
|  | Grade | 0.035 | 0.038 | 0.030 | 0.92 | [-0.039, 0.108] | 0.359 |  |  |
|  | Device availability | 0.003 | 0.018 | 0.006 | 0.16 | [-0.032, 0.038] | 0.875 |  |  |
|  | Internet quality | 0.002 | 0.017 | 0.005 | 0.14 | [-0.031, 0.036] | 0.887 |  |  |
|  | Parental education | 0.041 | 0.017 | 0.083 | 2.47 | [0.008, 0.074] | 0.014 |  |  |
|  | Digital learning duration | 0.014 | 0.017 | 0.027 | 0.82 | [-0.020, 0.048] | 0.410 |  |  |
|  | Digital readiness (T1) | 0.258 | 0.034 | 0.288 | 7.68 | [0.192, 0.324] | <0.001 |  |  |
|  | Family support (T1) | 0.106 | 0.029 | 0.123 | 3.64 | [0.049, 0.164] | <0.001 |  |  |
|  | Digital readiness (T1) × Family support (T1) | 0.253 | 0.043 | 0.191 | 5.85 | [0.168, 0.338] | <0.001 |  |  |
|  | Learning engagement (T1) | 0.123 | 0.032 | 0.129 | 3.80 | [0.059, 0.186] | <0.001 |  |  |

Note. *B* = unstandardized coefficient; *SE* = standard error; *β* = standardized coefficient. Continuous variables were mean-centered before the interaction term was created. *R*² and *F* are model-level statistics and are displayed in the first data row; the model-level *F* test was significant at *p* < 0.001. The total-effect and direct-effect models are reported in Table 4 of the main text.

**Table S7.** *Full simple slopes and conditional indirect associations.*

| **Effect** | **Estimate** | ***SE*/Boot *SE*** | ***t*** | **95% CI** | ***p*** |
| --- | --- | --- | --- | --- | --- |
| Simple slope at low family support (-1 *SD*) | 0.091 | 0.044 | 2.10 | [0.006, 0.177] | 0.036 |
| Simple slope at mean family support | 0.258 | 0.034 | 7.68 | [0.192, 0.324] | <0.001 |
| Simple slope at high family support (+1 *SD*) | 0.424 | 0.044 | 9.57 | [0.337, 0.511] | <0.001 |
| Conditional indirect association at low family support | 0.026 | 0.013 | — | [0.001, 0.053] | — |
| Conditional indirect association at mean family support | 0.073 | 0.013 | — | [0.048, 0.100] | — |
| Conditional indirect association at high family support | 0.119 | 0.019 | — | [0.084, 0.158] | — |
| Index of conditional indirect association | 0.071 | 0.014 | — | [0.045, 0.101] | — |

*Note.* Bootstrap confidence intervals were based on 5,000 resamples. *p* values are reported for simple slopes only; conditional indirect associations and the index were evaluated by bootstrap 95% CIs.

**Table S8.** *School-level ICCs and school-clustered robustness checks.*

| **Analysis** | **Predictor** | ***B*** | **Cluster-robust *SE*** | ***t*/ICC** | ***p*** |
| --- | --- | --- | --- | --- | --- |
| ICC: Digital self-efficacy (T2) | — | — | — | 0.000 | — |
| ICC: Learning engagement (T2) | — | — | — | 0.004 | — |
| Total-effect model | Digital readiness (T1) | 0.237 | 0.040 | 5.90 | <0.001 |
| Direct-effect model | Digital readiness (T1) | 0.166 | 0.040 | 4.13 | 0.001 |
| Direct-effect model | Digital self-efficacy (T2) | 0.282 | 0.033 | 8.53 | <0.001 |
| First-stage moderated model | Digital readiness (T1) | 0.258 | 0.023 | 11.07 | <0.001 |
| First-stage moderated model | Family support (T1) | 0.106 | 0.021 | 5.13 | <0.001 |
| First-stage moderated model | Digital readiness (T1) × Family support (T1) | 0.253 | 0.036 | 6.97 | <0.001 |

*Note.* Students were nested within 15 rural primary schools. ICCs were estimated for the two T2 outcomes. Cluster-robust standard errors were computed using school as the cluster variable. Because the number of clusters was limited, the school-clustered results should be interpreted as robustness checks rather than definitive multilevel evidence.
